# Thorough characterization of a new curvulavirid from a Japanese strain of Cryphonectria nitschkei

**DOI:** 10.1099/jgv.0.002177

**Published:** 2025-12-17

**Authors:** Sabitree Shahi, Sakae Hisano, Wasiatus Sa'diyah, Yoshihiro Takaki, Hideki Kondo, Nobuhiro Suzuki

**Affiliations:** 1 Institute of Plant Science and Resources, Okayama University, Kurashiki, Okayama 710-0046, Japan; 2 Institute for Extra-cutting-edge Science and Technology Avant-garde Research (X-star), Japan Agency for Marine-Earth Science and Technology (JAMSTEC), Yokosuka, Japan; 3 Neo-Virology Laboratory, Graduate School of Agricultural Science, Tohoku University, Sendai, Miyagi 980-8572, Japan

**Keywords:** curvulavirus, *Cryphonectria carpinicola*, *Cryphonectria nitschkei*, *Cryphonectria parasitica*, fungal dsRNA virus, host range, RNA silencing

## Abstract

A new curvulavirid was isolated from a Japanese strain of the filamentous ascomycete Cryphonectria nitschkei and thoroughly characterized. The virus termed Cryphonectria nitschkei curvulavirus 1 (CnCvV1) has a bi-segmented dsRNA genome. CnCvV1 dsRNA1 encodes an RNA-dependent RNA polymerase (592 amino acids), while dsRNA2 possesses two ORFs, one that encodes a protein associated with the genomic dsRNA and the other that encodes a hypothetical protein of unknown function. CnCvV1 could be experimentally introduced into another virus-free strain of *C. nitschkei* and two strains of different fungal species within the genus *Cryphonectria* (*Cryphonectria parasitica* and *Cryphonectria carpinicola*). Based on phenotypic comparison, the virus caused asymptomatic infection in the three newly established fungal strains. However, there was a reduced colony growth rate and increased CnCvV1 accumulation in an RNA silencing-deficient mutant (Δ*dcl2*), relative to the wt strain EP155 of a model virus host fungus (*C. parasitica*). These findings suggest that CnCvV1 is targeted by RNA silencing in *C. parasitica*. This study provides a foundation for further exploration of curvulavirids that have been biologically understudied.

## Data Summary

The complete nucleotide sequences of two CnCvV1 strains in this article have been deposited with the EMBL/GenBank/DDBJ Data Library under accession nos. LC781670-LC781673.

## Introduction

The phylum *Pisuviricota* is unique because it contains both single-stranded (ss) RNA and double-stranded (ds) RNA viruses [1]. Members with dsRNA genomes, which include partitivirids, amalgavirids and picobirnavirids, are classified in the order *Durnavirales*. The family *Curvulaviridae* has recently been established in *Durnavirales* and possesses one genus, *Orthocurvulavirus*, which currently includes eight species [2]. An increasing number of orthocurvulavirus sequences have been reported. These viruses have been detected from diverse filamentous fungi, such as a sea cucumber-associated marine ascomycete [3]; endophytic, phytopathogenic and entomopathogenic ascomycetes [4,8]; and phytopathogenic basidiomycetes [9,10]. These viruses are often named ‘… bipartite virus’ after their predicted bipartite nature. There are two common genome features: a bi-segmented dsRNA genome with a terminally conserved sequence and a larger segment, dsRNA1, that encodes RNA-dependent RNA polymerase (RdRP). However, there are also dissimilarities among them. The genomic segments of some curvulavirids have been reported to have two open reading frames (ORFs), while those of others have only one.

Although the genome sequence of many classified and unclassified orthocurvulaviruses is available, there is scarce biological information about them except for that of Curvularia thermal tolerance virus (CThTV) and Heterobasidion RNA virus 6 (HetRV6). Interestingly, the CThTV-harbouring host ascomycetous fungus, *Curvularia protuberata,* appears to confer heat tolerance to the plants able to host the fungus naturally and even experimentally [8,11]. CThTV was the first identified orthocurvulavirus that has a bi-segmented dsRNA genome, with each segment having two ORFs [8]. HetRV6 exerts varying phenotypic effects – from harmful to beneficial – on the host fungus fitness depending on host species and environmental conditions [12]. There are some similarities between CThTV and partitiviruses, such as a bi-segmented dsRNA genome, genome size and isometric particle morphology [8,13, 14]. The second ORF of CThTV dsRNA1 encodes RdRP. However, gene-product assignment is unavailable for the other ORFs. There is also ambiguity regarding the encapsidation nature of curvulavirids. CThTV is the only curvulavirid for which spherical particles are established. Notably, HetRV6 RdRP expressed in *Escherichia coli* has been shown to catalyse the *in vitro* synthesis of dsRNA in a primer-dependent, sequence-independent manner using ssRNA as a template [15].

The fungal genus *Cryphonectria* (Cryphonectriaceae, Diaporthales and Ascomycota) includes important phytopathogenic fungi such as *Cryphonectria parasitica*, which causes one of the three most destructive tree diseases [16,18]. Notably, *C. parasitica* serves as a virus host model of filamentous fungi in which virus–host interactions can be explored using a variety of viruses [19,20]. In particular, the virus–*C. parasitica* system has been used to study antiviral RNA silencing and counter-defence [21,26]. In contrast to *C. parasitica*, other *Cryphonectria* species have not been explored as virus hosts, and several viruses have been identified from them [27,29]. An example is *Cryphonectria nitschkei*, also known as *Cryphonectria japonica*; it is closely related to *C. parasitica* and sympatric with it on chestnut trees (genus *Castanea*) [30,31] but much less pathogenic to chestnut trees [17]. Milgroom *et al*. [31] reported viruses detected from Japanese isolates of *C. nitschkei*. The Research Center of Genetic Resources, National Agriculture and Food Research Organization (NARO), Japan (https://www.gene.affrc.go.jp/about_en.php), has a relatively large collection of field-collected isolates of *C. nitschkei*. We started a screen of the collection for viruses and previously reported characterization of a chrysovirus (order *Ghabrivirales*) with a dsRNA genome [32].

This study is an extension of the virus-hunting study using a *C. nitschkei* collection and presents a thorough characterization of a novel curvulavirid termed Cryphonectria nitschkei curvulavirus 1 (CnCvV1). Specifically, we determined the genome organization, symptomless nature and relatively narrow host range of CnCvV1. Furthermore, we showed that CnCvV1 is targeted by RNA silencing or RNAi in the model filamentous fungus, *C. parasitica*.

## Methods

### Fungal strains

The *C. nitschkei* strains LFP-E24 (E24, MAFF 410076), E16 (MAFF 410155) and E49 (MAFF 410604) were purchased from the Ministry of Agriculture, Forestry and Fisheries (MAFF), Japan (https://www.gene.affrc.go.jp/index_j.php). *C. nitschkei* E24 was originally isolated from *Quercus mongolica* var. *grosserrata* (syn. *Q. cryspula*); we previously tested its ability to host a chrysovirus [32]. *C. nitschkei* E16 and E49 were originally collected from *Quercus serrata* and *Q. mongolica* var. *grosserrata*, respectively. The fungal strains were grown on Difco potato dextrose agar (PDA) (Becton, Dickinson and Co., Franklin Lakes, NJ). All fungal strains used in this study are listed in Table 1.

**Table 1.** Fungal and viral strains used in this study

Family/species	Strain	Original host plant or description	Reference or source
Cryphonectriaceae			
*Cryphonectria nitschkei*	LFP-E24 (E24)	Japanese oak (*Quercus mongolica* var. *grosserrata*), strain infected by CnCvV1	MAFF 410076
	E24-Mix	E24 (natural CnCvV1 infectant) coinfected by CHV1 and CnCvV1	[32]
	E16	Jolcham oak (*Q. serrata*)	MAFF 410155
	E49	Japanese oak (*Q. mongolica var*. *grosserrata*), strain infected by CnCvV1	MAFF 410604
*C. parasitica*	EP155	American chestnut (*Castanea dentata*), standard fungal strain	ATCC 38755 [33]
	ΕΠ155/CHV1	EP155 infected by CHV1-EP713	[73]
	Δ*dcl2*	RNA silencing-deficient mutant of *C. parasitica* strain EP155	[21]
*C. carpinicola*	JS13	Cork oak (*Quercus suber*)	[29,31]
Valsaceae			
*Valsa ceratosperma*	AVC53	Apple tree (*Malus domestica*)	[34]
Nectriaceae			
*Fusarium oxysporum*	7n	Tomato in a vegetable market, strain infected by hadakavirus 1 (HadV1)	[46]
Aspergillaceae			
*Penicillium janthinellum*	A58	Tobacco–potato double-cropping soil, strain infected by Penicillium janthinellum polymycovirus 1 (PjPmV1)	[51]

*C. parasitica* EP155, a standard strain; *C. parasitica* Δ*dcl2*, Dan RNA silencing-deficient mutant [21,33]; and a Japanese strain (AVC53) of *Valsa ceratosperma*, the causal fungus of apple Valsa canker [34], were generously provided by Drs. Donald L. Nuss (Institute for Bioscience and Biotechnology Research, University of Maryland) and Satoko Kanematsu (NARO, Japan).

### Sequence determination of CnCvV1

The CnCvV1 genome sequence was determined based on a combined approach with next-generation sequencing (NGS) and Sanger sequencing of reverse transcription (RT)-PCR clones. For NGS, total RNA fractions (see below) were obtained from *C. nitschkei* E24 and sent to Macrogen Inc. (Tokyo, Japan) for rRNA depletion and cDNA library generation as described by Kondo *et al*. [35]. Contig sequences assembled by CLC Genomics Workbench (version 22, CLC Bio-QIAGEN, Aarhus, Denmark) were subjected to local blast searches for sequence similarities with virus reference sequences (RefSeq) deposited in the National Center for Biotechnology Information (NCBI) database. The terminal sequences of viral segments were determined by the 3′ RNA ligase-mediated rapid amplification of cDNA ends (RLM-RACE) procedure as described by Suzuki *et al*. [36]. Reverse transcription PCR was performed to amplify the viral genomic sequences obtained by NGS. RLM-RACE and RT-PCR clones were subjected to Sanger sequencing.

The nucleotide sequences of dsRNA1 and dsRNA2 of CnCvV1 are deposited in the GenBank/DDBJ/EMBL database under accession nos. LC781670 and LC781671, respectively, for *C. nitschkei* E24 and LC781672 and LC781673, respectively, for *C. nitschkei* E49. The oligonucleotides used in RACE and RT-PCR are listed in Table S1, available in the online Supplementary Material.

### Sequence and phylogenetic analyses

The CnCvV1 sequences were analysed by using the sequence processing software GENETX ver. 20 (GENETYX, Tokyo, Japan). The blast program available from NCBI (nucleotide or protein collection, http://blast.ncbi.nlm.nih.gov/Blast.cgi) was used to search for viral sequences. To study the relationship between CnCvV1 and orthocurvulaviruses, a maximum likelihood phylogenetic analysis was performed using RdRP, following a previously described method [37]. Multiple amino acid sequences were aligned using MAFFT version 7 (https://mafft.cbrc.jp/alignment/server/). Unreliable regions in the alignment were trimmed using trimAl version 1.3 in the Phylemon 2.0 online platform, with the automated 1 setting (http://phylemon.bioinfo.cipf.es) [38]. Then, the IQ-TREE web server platform [39] was used to select the best-fitting model via ModelFinder [40] and to construct the phylogenetic tree, supported by SH-aLRT/ultrafast bootstrap analysis [41]. The resulting phylogenetic tree was visualized using the FigTree software version 1.3.1 (https://nucleobytes.com/enzymex/index.html) with midpoint rooting.

### Protoplast fusion and transformation

Fungal strains were grown in potato dextrose broth (PDB) (Becton, Dickinson and Co.) and used for protoplast preparation as described by Churchill *et al*. [42]. An equal number of protoplasts obtained from a donor and recipient strain (~1×10^7^) were fused in the presence of polyethylene glycol as described by Shahi *et al*. [26]. The recipient strains had previously been tagged with a hygromycin resistance gene (hygromycin B phosphotransferase) as a selection marker [26,32].

### RNA preparation and analyses

Total ssRNA fractions were obtained by using the method described by Eusebio-Cope and Suzuki [43] and subjected to NGS and northern blotting. Total RNA (2 µg) was heat denatured and electrophoresed in a 1% agarose gel under denaturing conditions. The RNA separated in the gel was blotted onto a Hybond-N^+^ nylon membrane (GE Healthcare Life Sciences, Pittsburgh, PA, USA) and probed with digoxigenin (DIG)-labelled DNA amplified from cDNA by PCR (PCR DIG Labelling Mix, Roche, Risch-Rotkreuz, Switzerland) after fixation with a UVP CL-1000 UV crosslinker (UVP Analytik Jena GmbH+Co., Jena, Germany). Then, the membrane was subjected to the prehybridization and hybridization steps, which were performed based on the information provided by the supplier (Roche). Specific RNA bands were detected by using ready-to-use CDP-Star (Roche) through digital imaging in the ImageQuant LAS 4000 system (GE Healthcare Life Sciences).

Reverse transcription quantitative PCR (qPCR) was basically performed as described by Sato *et al*. [44] using primers listed in Table S1. The sequences of *dcl2* mRNA are publicly unavailable for *C. nitschkei* and *Cryphonectria carpinicola*. To design appropriate primers for their *dcl2* mRNA, cDNA fragments covering map positions 1,996 to 2,754 in *dcl2* transcripts of *C. parasitica* [21] were first amplified using total RNA from *C. nitschkei* and *C. carpinicola*. A primer pair EP-m-dcl2-1996F and EP-m-dcl2-2754R (Table S1) was designed based on the sequence of C. parasitica *dcl2* mRNA. After Sanger sequencing amplified cDNA fragments, another pair of primers (EP-g-dcl2-2119F and EP-g-dcl2-2297R) was prepared based on portions strictly conserved across three fungal species. Actin (*act*) mRNA was used as an internal control.

 Direct colony reverse transcription PCR was carried out as described earlier [45,46] using the PrimeScript™ One Step RT-PCR Kit ver. 2 (Dye Plus, Takara Bio, Inc., Kusatsu, Japan).

### Virion purification, protein analyses and transfection

 Crude virus particle fractions were prepared from *C. nitschkei* E24, naturally infected by CnCvV1, based on a conventional method described by Sato *et al*. [46] with minor modifications. Particles were enriched by high-speed centrifugation of the homogenate after mixing with 8% (w/v) polyethylene glycol (PEG; Mw. 6000) and 1% (w/v) NaCl for 2 h on ice. The particle suspension was further subjected to CsCl equilibrium and sucrose gradient centrifugation to examine for dsRNA by agarose gel electrophoresis and protein components by 10% SDS-PAGE.

Crude particle fractions and particle fractions after CsCl and sucrose gradient centrifugation were used to transfect protoplasts derived from a few virus-free fungal strains of different fungal species listed in Table 1, with the method described by Chiba *et al*. [47]. Protoplasts were prepared from fungal mycelia cultured in PDB (Becton, Dickinson and Co.) liquid medium as described by Eusebio-Cope and Suzuki [43].

Peptide mass fingerprinting (MS/MS analysis) of CnCvV1 36-kDa protein (p36) in virus-enriched fractions was performed at the Department of Instrumental Analysis and Cryogenics, Advanced Science Research Center, Okayama University (Okayama, Japan). Proteins in CnCvV1 fractions were separated in a 10% SDS-PAGE gel, which was stained with the Rapid Stain CBB Kit (Nacalai Tesque Inc., Kyoto, Japan) or the SilverQuest Silver Staining Kit (Thermo Fisher Scientific Inc., Waltham, MA, USA). CnCvV1 36-kDa protein (p36) was digested in-gel by trypsin (Bruker Daltonics Inc., Billerica, MA, USA). The digested peptides were analysed with the HPLC-Chip/QTOF system (Agilent Technologies Inc., Santa Clara, CA, USA) at the Department of Instrumental Analysis and Cryogenics, Okayama University, and identified using the Mascot software (Matrix Science Inc., Boston, MA, USA).

### Electron microscopy

Purified CnCvV1 fractions obtained as described above were stained with EM stainer (Nissin EM Co., Tokyo, Japan), which is an alternative to uranyl acetate [48]. The stained specimens were observed with an H-7650 transmission electron microscope (Hitachi, Tokyo, Japan).

### RNase A assay

The RNase assay was conducted on ice or at 37 °C as described by Sato *et al*. [46]. Crude extracts of virus particles, containing not only virus particles but also nucleic acids, were prepared from different fungal strains infected by different viruses. Approximately 500 mg of cellophane-PDA cultured mycelia was powdered in liquid nitrogen, homogenized in 5 ml of 0.1 M sodium phosphate (pH 7.0) and centrifuged twice (4,000 r.p.m. for 10 min each time) to remove the cell debris. The supernatant was divided into four tubes, each containing 0.9 ml, and kept on ice. RNase A (Sigma-Aldrich Co. LLC, St. Louis, MO, USA) in 100 µl was added at a final concentration of 10 µg ml^−1^ and incubated at 37 °C with or without 1% (v/v) Triton X-100. After the incubation, ethylenediaminetetraacetic acid was added to a final concentration of 1 mM to inhibit RNase. The dsRNA fractions were extracted by phenol, phenol/chloroform, chloroform/isoamyl-alcohol treatments and cellulose column purification. The resulting dsRNA was examined by running the entire sample on an agarose gel.

## Results

### Organization of the CnCvV1 genome

**Fig. 1.**
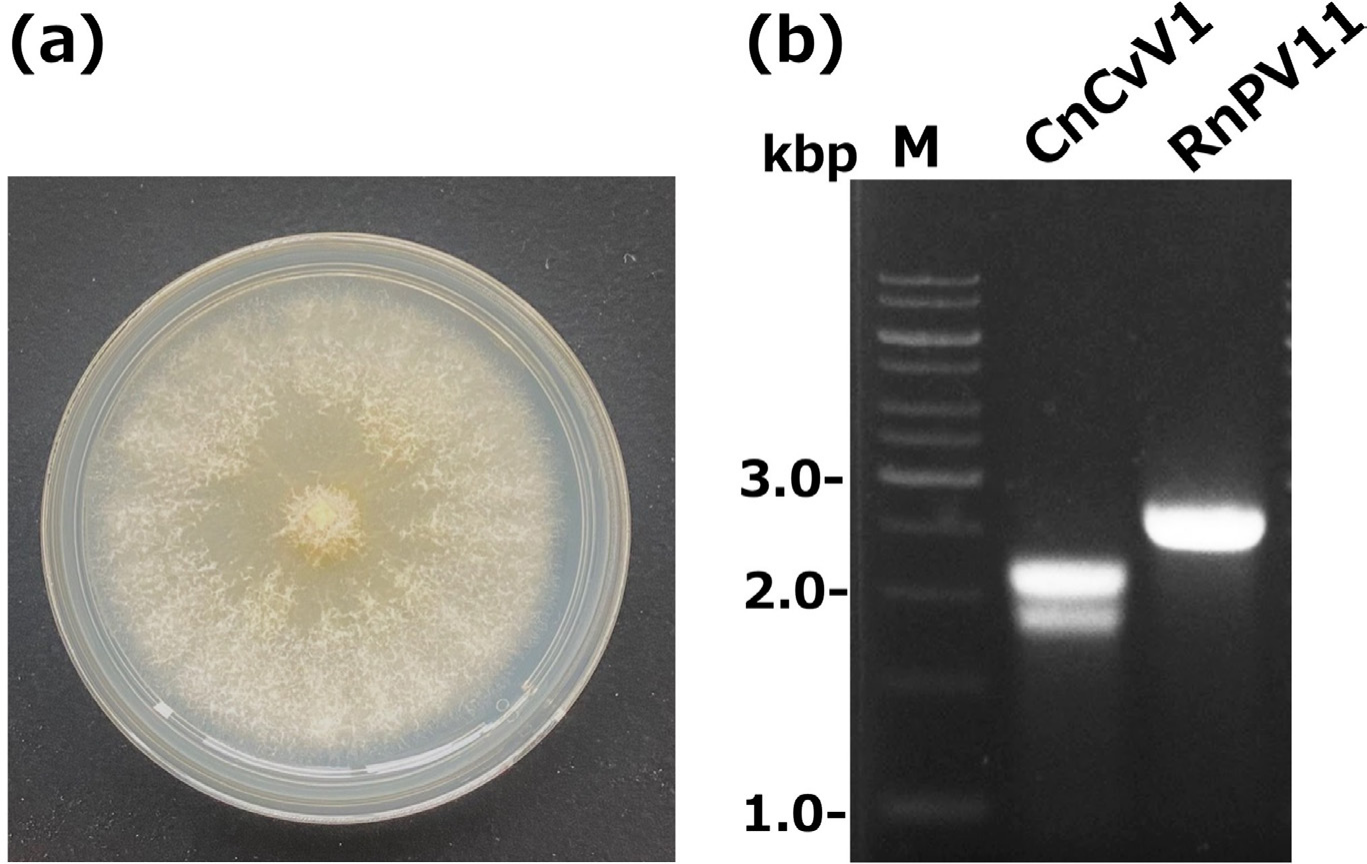
Colony morphology of the *C. nitschkei* strain harbouring CnCvV1. (**a**) Colony phenotype of the CnCvV1-infected *C. nitschkei* E24. The fungus was grown on PDA plates for 1 week at room temperature (~25 °C). (**b**) The dsRNA pattern of CnCvV1-infected *C. nitschkei* E24. The dsRNA fractions were prepared from mycelia grown in PDB and electrophoresed in a 1.0% agarose gel in TAE buffer. dsRNA purified from *Rosellinia necatrix* W98 infected by a partitivirus (RnPV11) [74] was electrophoresed in parallel. M refers to the 1 kb dsDNA ladder size marker (GeneRuler 1 kb DNA ladder, Thermo Fisher).

We screened over 20 Japanese strains of *Cryphonectria* spp. for virus infections, among which were two *C. nitschkei* strains, E24 (Fig. 1a) and E49 (data not shown), which carried dsRNA detectable by the agarose gel assay (Fig. 1b, lane CnCvV1). We obtained the NGS data from two pools of total RNA obtained from seven and two fungal strains of *Cryphonectria* spp., respectively (see the ‘Methods’ section). The NGS data showed the presence of several mycoviruses in the pooled RNA. Note that, in this study, we only report two CnCvV1 strains from *C. nitschkei* E24 and E49, the total RNA of which was included in the different pools. We will report other virus sequences detected in the two pools in future studies. We obtained a total of four curvulavirid contigs, each over 1,000 nt in length. Colony reverse transcription PCR confirmed infection of the two fungal strains by CnCvV1 (data not shown). We determined the terminal sequences of each CnCvV1 isolate by performing RLM-RACE followed by Sanger sequencing, which confirmed the NGS-generated contig sequences. The accession numbers of the newly determined complete sequences for the two CnCvV1 strains are LC781670 and LC781671 (*C. nitschkei* E24) and LC781672 and LC781673 (*C. nitschkei* E49).

**Fig. 2.**
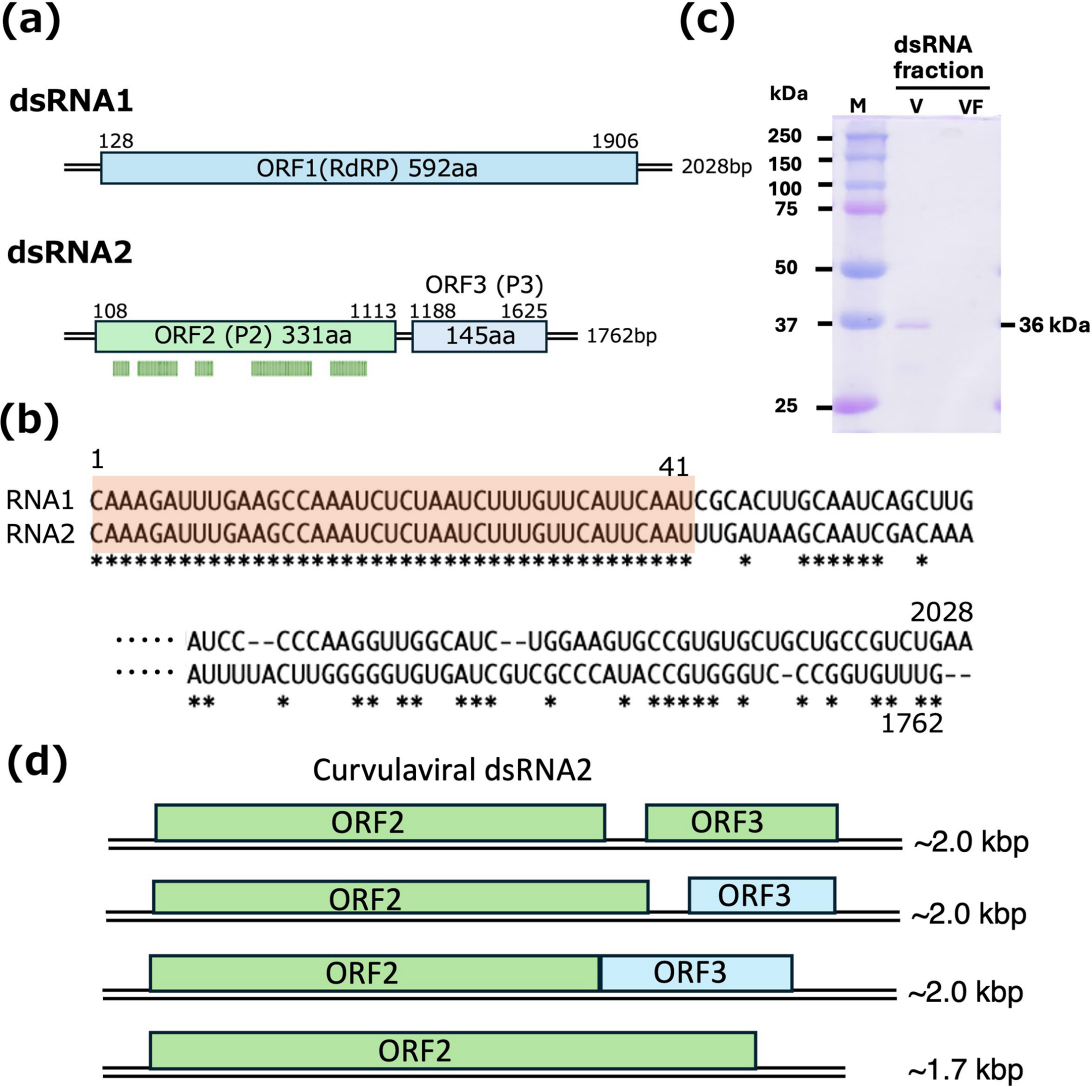
Genome organization and protein components of CnCvV1. (**a**) Schematic representation of the CnCvV1 genomic dsRNA segments. The segment length (in bp) is shown on the right. The ORFs for each genomic segment are shown by coloured boxes. The position of the start/stop codon for the ORFs is indicated on each ORF of dsRNA1 and dsRNA2. Mapping of the coding region of CnCvV1 dsRNA-associated protein was conducted following in-gel tryptic digestion of p36 (P2) (**c**) and liquid chromatography–MS/MS analysis. Mapped peptide fragments derived from virus fractions (**c**) are indicated by green bars below ORF2 (P2). (**b**) Comparison of the terminal sequences of CnCvV1 strains. The terminal sequences are conserved between the three CnCvV1 strains: CnCvV1-E24 (LC781670 and LC781671), CnCvV1-E49 (LC781672 and LC781673) and Cryphonectria parasitica bipartite virus 1 (KC549809 and KC549810). The 5′-terminal 41-nt stretch is strictly conserved between the two genomic segments of each viral strain (highlighted in beige), whereas no such conserved sequence stretch is detected at the 3′-termini. (**c**) Protein components of a fraction obtained by a conventional sucrose gradient centrifugation method for the spherical particle purification. Virus fractionation was conducted for *C. nitschkei* E24. A CnCvV1-dsRNA-enriched fraction was denatured at 95 °C for 5 min in the presence of sodium dodecyl sulphate and *β*-mercaptoethanol and electrophoresed on a 10% polyacrylamide gel (**v**). Proteins were stained with Coomassie Brilliant Blue R250. A fraction obtained from virus-free *C. nitschkei* E16 by the same method was treated in parallel (VF). M refers to the protein size marker (Precision Plus Protein Dual Colour Standards, Bio-Rad Laboratories, Inc., Hercules, CA, USA). (**d**) ORF configuration of dsRNA2 in curvulavirids. Four types of dsRNA2 ORF organizations were identified among the curvulavirids included in the phylogenetic analysis shown in Fig. 3 (see below). ORF3 is located in the same reading frame as ORF2 (top) in most curvulavirids; in a different reading frame (second from top) in a few curvulavirids, represented by *Curvularia thermal* tolerance virus and Fusarium graminearum dsRNA mycovirus 4; overlapping with ORF2 (either in-frame or out-of-frame) (second from bottom) in several curvulavirids such as Heterobasidion RNA virus 6; or absent (bottom) in some curvulavirids, including Rhizoctonia solani dsRNA virus 1.

The genome organization of the CnCvV1 strain based on the confirmed sequences is shown in Fig. 2a. The larger segment, dsRNA1, is 2,028 bp in length and encodes a putative RdRP, while the smaller segment, dsRNA2, is 1,762 bp and contains two ORFs (ORF2 and ORF3) that could encode 331 and 145 aa, respectively.

blastp analysis with CnCvV1 RdRP showed hits to many other curvulavirids. Of note, CnCvV1 RdRP exhibits 98.5% sequence identity with that of another curvulavirid termed Cryphonectria parasitica bipartite mycovirus 1 (CpBMV1) reported by a Chinese group led by Dr. Daohong Jiang (accession number AGK89731) isolated from a different fungal species, *C. parasitica*. Other than its genome sequence, no information about CpBMV1 is available. The second-highest RdRP sequence identity (65.4%) is with Diaporthe eres bipartite mycovirus 1 (DeBV1) (72.5%, XQH47654.1), followed by Botryosphaeria dothidea bipartite mycovirus 1 (BdBV1) (65.4%) [5]. RdRPs from other curvulavirids, such as Botrytis cinerea mycovirus 3 [6], also share moderate sequence identity with CnCvV1 RdRP.

The dsRNA2 segment of most curvulavirids has two separate ORFs: ORF2 (P2) and ORF3 (P3). ORF2 appears to encode a capsid protein and is conserved among curvulavirids. CThTV capsid protein was predicted to be encoded by dsRNA2 ORF2 based solely on the molecular mass of the capsid protein [8]. CnCvV1 P2, with the highest sequence identity (99.1%) to CpBMV1 P2 (AGK89732), associated with the genomic dsRNA (see below), exhibited 57.0% and 50.7% sequence identity to the homologous proteins of DeBV1 and BdBV1, respectively. Curvulavirid dsRNA2 ORF3 with a similar coding capacity of 90~150 aa is observed in a similar position of the dsRNA2 segment – that is, downstream of ORF2 in a different frame (data not shown). While an ORF3-encoded hypothetical protein of unknown function is presumably expressed, blastp did not reveal sequence similarity to known proteins. Note that we did not observe sequence similarity with an optimal score over 75, even among curvulavirus ORF3-encoded proteins when using pairwise sequence comparison with the GENETYX software. An exception is the CpBMV1 ORF3-encoded protein P3, which shows 95% sequence identity with an optimal score of 659.

The terminal sequences of the coding strands are 5′-CAAAGAUUUGAA---(ORF1)---UGCUGCCGUUCG-3′ for dsRNA1 and 5′-CAAAGAUUUGAA---(ORF2/3)---UCCCGGUGUUUG-3′ for dsRNA2 (Fig. 2b); they are shared with those of CpBMV1. Note that the 5′-terminal 41-nt stretch is strictly conserved (beige-highlighted), while the 3′-terminal sequences are not conserved. The conserved nature of only the 5′-terminal sequences between the two genomic segments of a curvulavirid has also been noted in other members of the family *Curvulaviridae* [7,49]. This finding contrasts with the conserved nature of both the terminal sequences among all the genomic segments of dsRNA viruses (e.g. chrysoviruses and partitiviruses) [14,50].

### Phylogenetic analysis of CnCvV1

**Fig. 3.**
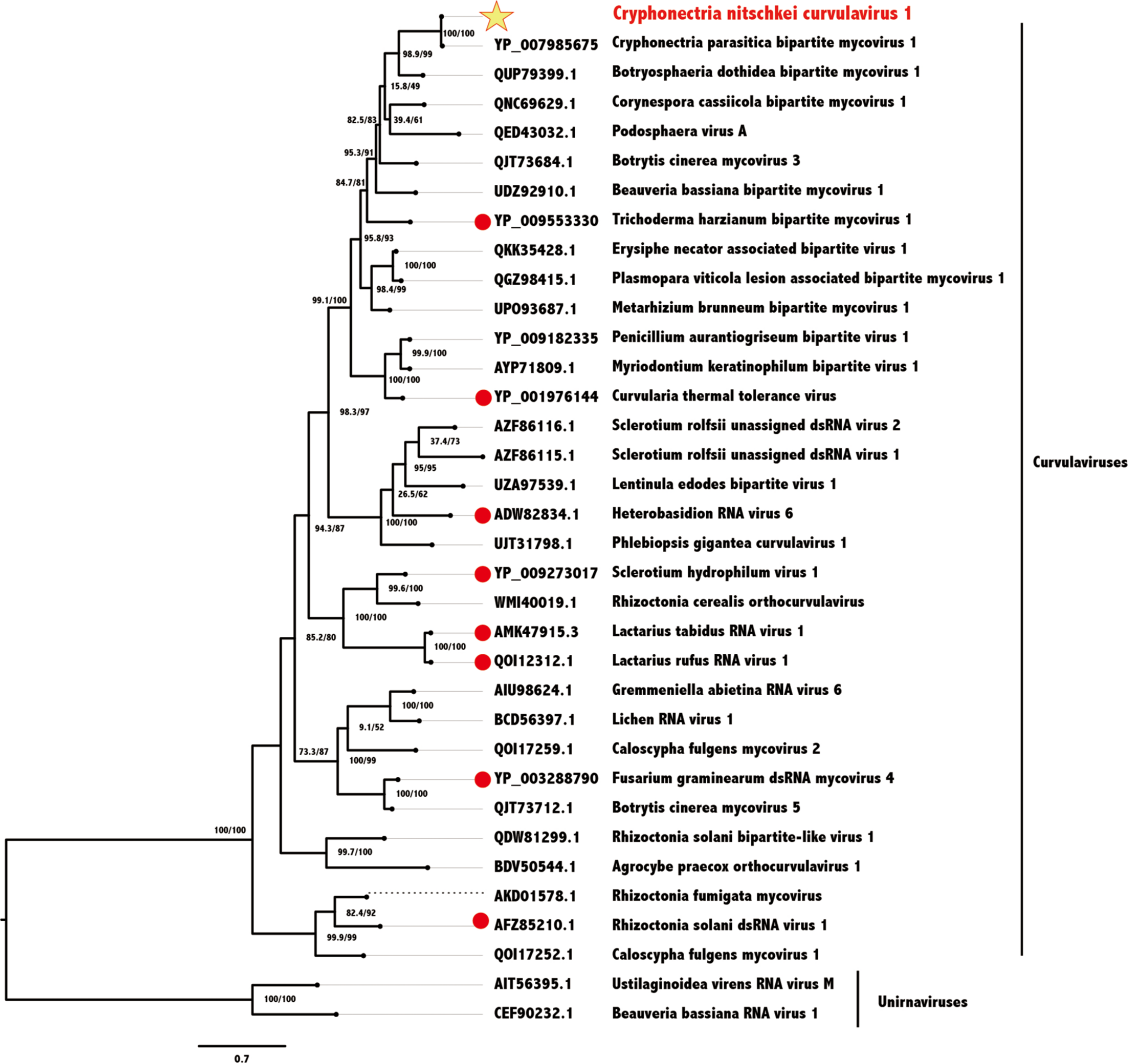
Phylogenetic relationships of CnCvV1 and orthocurvulaviruses. A maximum likelihood tree was constructed from a multiple sequence alignment of viral RdRP amino acid sequences. The virus names are preceded by their accession numbers. Members of the established virus species are indicated by red circles. CnCvV1 is marked with the yellow star. Two fungal unirnaviruses (Ustilaginoidea virens RNA virus M and Beauveria bassiana RNA virus 1) were used as outgroups. Branch numbers indicate SH-aLRT support (%)/ultrafast bootstrap support (%) values. Bars represent amino acid divergence.

We performed phylogenetic analyses based on the predicted amino acid sequences of the RdRP (encoded by dsRNA1) of orthocurvulaviruses (family *Curvulaviridae*, order *Durnavirales*). CnCvV1 and its potential variant, CpBMV1, form a subcluster with a known member, Trichoderma harzianum bipartite mycovirus 1, and several unassigned orthocurvulaviruses (Fig. 3). Among these, CnCvV1 is most closely related to BdBV1, sharing 65% amino acid sequence identity.

### Attempts to purify CnCvV1 particles and to characterize protein components

We attempted to purify CnCvV1 particles with the conventional methods (see the ‘Methods’ section) for isometric fungal dsRNA viruses such as megabirnaviruses and quadriviruses. Although we obtained fractions enriched with genomic RNA and a 36 kDa protein (p36) (Fig. 2c), we could not find spherical particles by electron microscopy, which we expected based on another curvulavirid, CThTV. We confirmed that p36 is the ORF2-encoded protein P2 based on peptide mass fingerprinting (Fig. 2a). P2 appeared to be associated with the genomic dsRNA, as it copurified with its genomic dsRNA (Figs 2c and S1). However, CnCvV1 fractionation patterns were distinct from typical isometric particles such as victoriviruses and partiviruses, which were isolated in fractions 8~11 in both gradients under the same centrifugation conditions (Fig. S1).

**Fig. 4.**
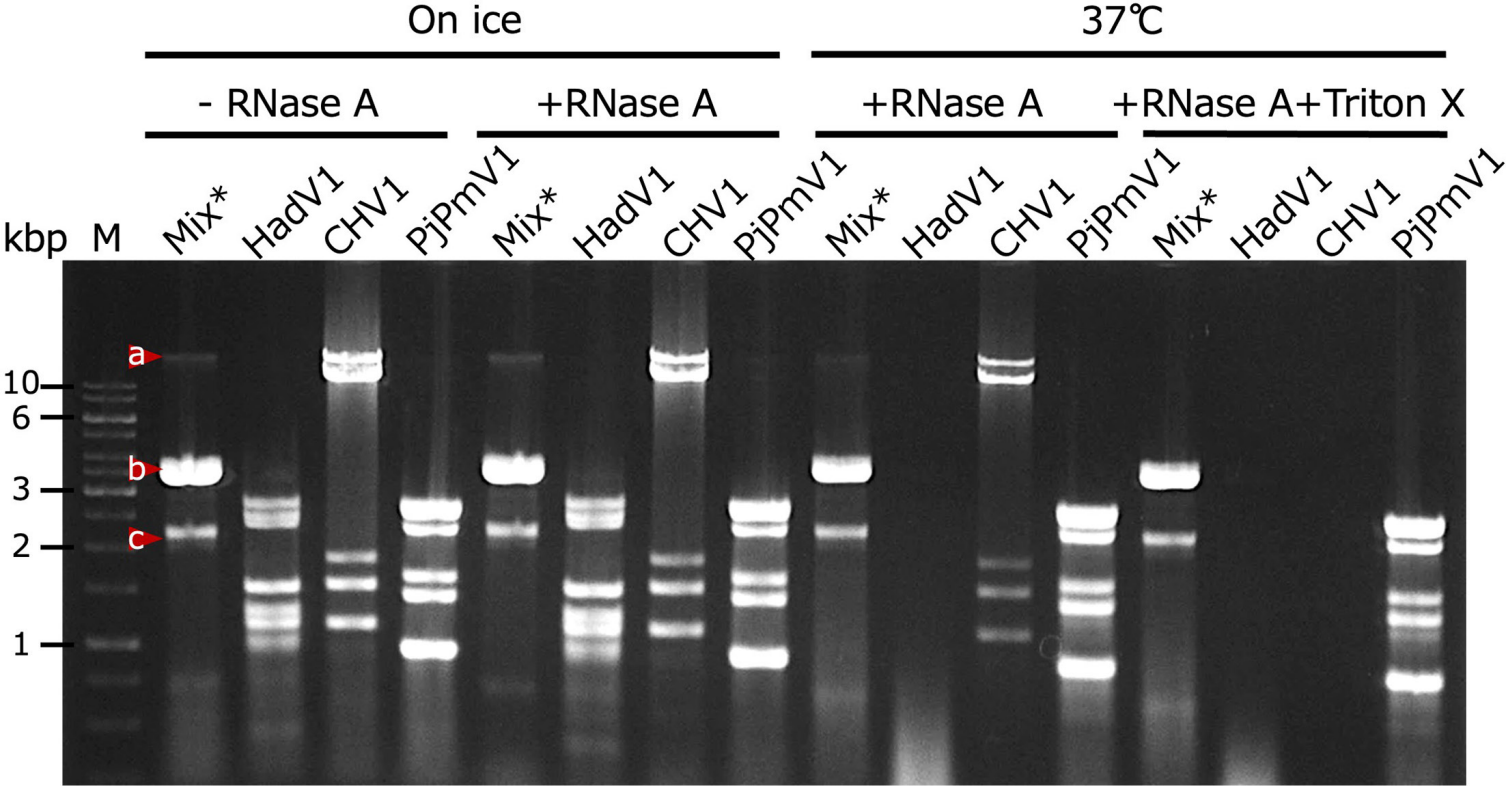
The effects of RNase treatment on different viral genomic dsRNA or replicative form dsRNAs. RNase A treatment (with different conditions as described in the ‘Methods’) was used to examine the virus entity in host mycelial homogenates. Four fungal strains were utilized: *C. nitschkei* E24 (E24-mix, Mix*) coinfected by capsidless CHV1 (band a), encapsidated Cryphonectria nitschkei chrysovirus 1 (CnCV1, band b) and CnCvV1 (band c) [32]; *Fusarium oxysporum* 7n infected by naked HadV1 [46]; *C. parasitica* EP155 infected by CHV1 [73]; and *Penicillium janthinellum* A58 infected by the capsidless polymycovirus PjPmV1 [51]. See Table 1 for the fungal strains used in this study. The double-stranded RNA (dsRNA) fractions treated with RNase A were examined by 1% agarose gel electrophoresis. The red rectangle frames indicate CnCvV1 genomic dsRNA, while the blue frames denote the CHV1 replicative form dsRNA in E24-mix. The GeneRuler 1 kb DNA ladder (Thermo Fisher) was electrophoresed in parallel as the size standard (lane M).

 This fractionation profile of CnCvV1 prompted us to conduct an RNase A assay, which could help us determine the form of CnCvV1 in infected cells. We found that CnCvV1 dsRNA is resistant to RNase A even in the most digestible conditions: the presence of Triton X-100 at 37 °C, which digested capsidless hypovirus (CHV1) and hadakavirus (HadV1) dsRNA replicative forms (Fig. 4). Note that while hadakavirus is susceptible to RNase A at 37 °C in the absence of Triton X-100, hypoviruses became susceptible to it only in the presence of Triton X-100. The digestion pattern of CnCvV1 dsRNA is quite similar to that of a polymycovirus, PjPmV1, whose genomic dsRNA is tightly associated with its PASrp (proline-alanine-serine-rich protein) [51].

### Experimental host range expansion and horizontal transmission of CnCvV1

**Fig. 5.**
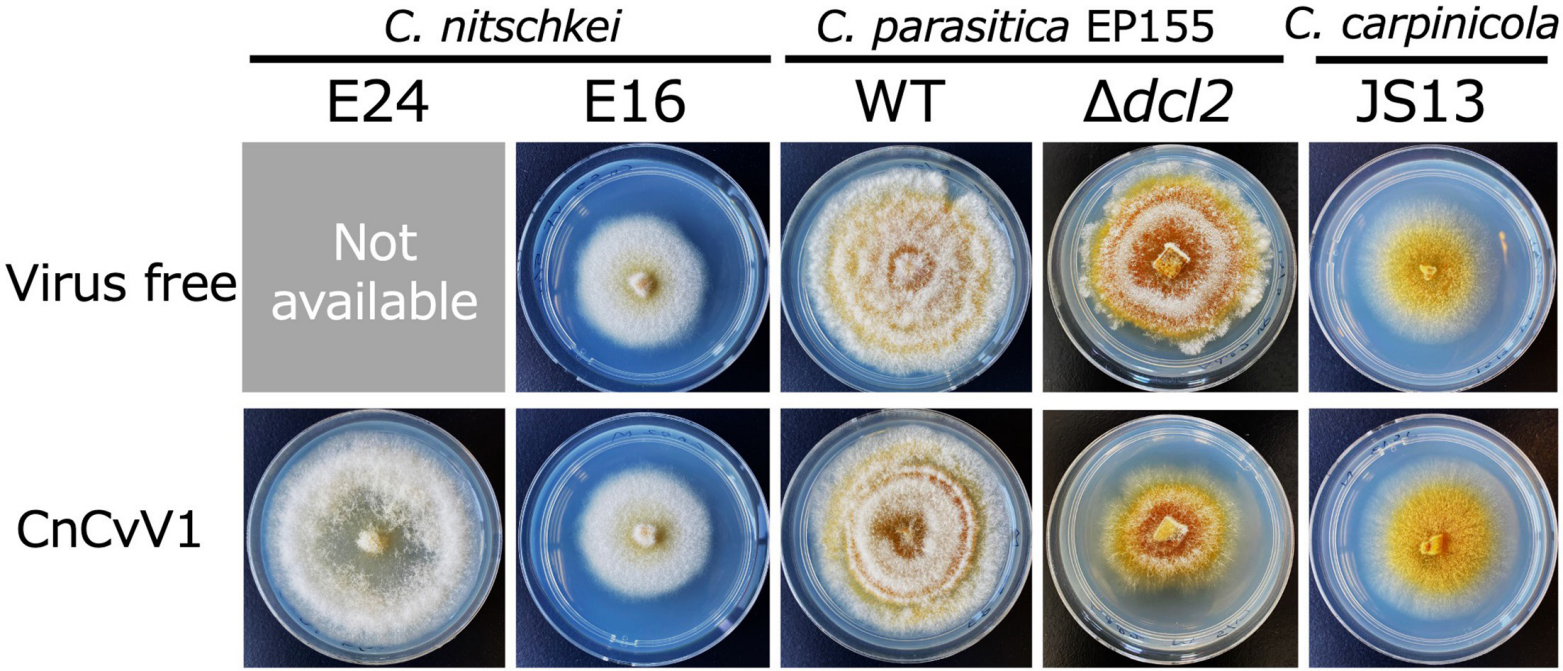
Phenotype of CnCvV1-infected host fungi. The colony morphology of four sets of CnCvV1-free and CnCvV1-infected isogenic fungal strains. CnCvV1 was transferred via protoplast fusion (Table 2) to four fungal strains: *C. nitschkei* E16, *C. parasitica* EP155 (wt) and Δ*dcl2* (an RNA silencing-deficient mutant derived from EP155) and *C. carpinicola* JS13. Four sets of the fungal strains were grown for 5 days on the benchtop and photographed. The original CnCvV1-infected *C. nitschkei* E24 was used as a reference. Note that *C. nitschkei* E24 could not be cured of CnCvV1. See the Fig. 1 legend for culture conditions.

**Fig. 6.**
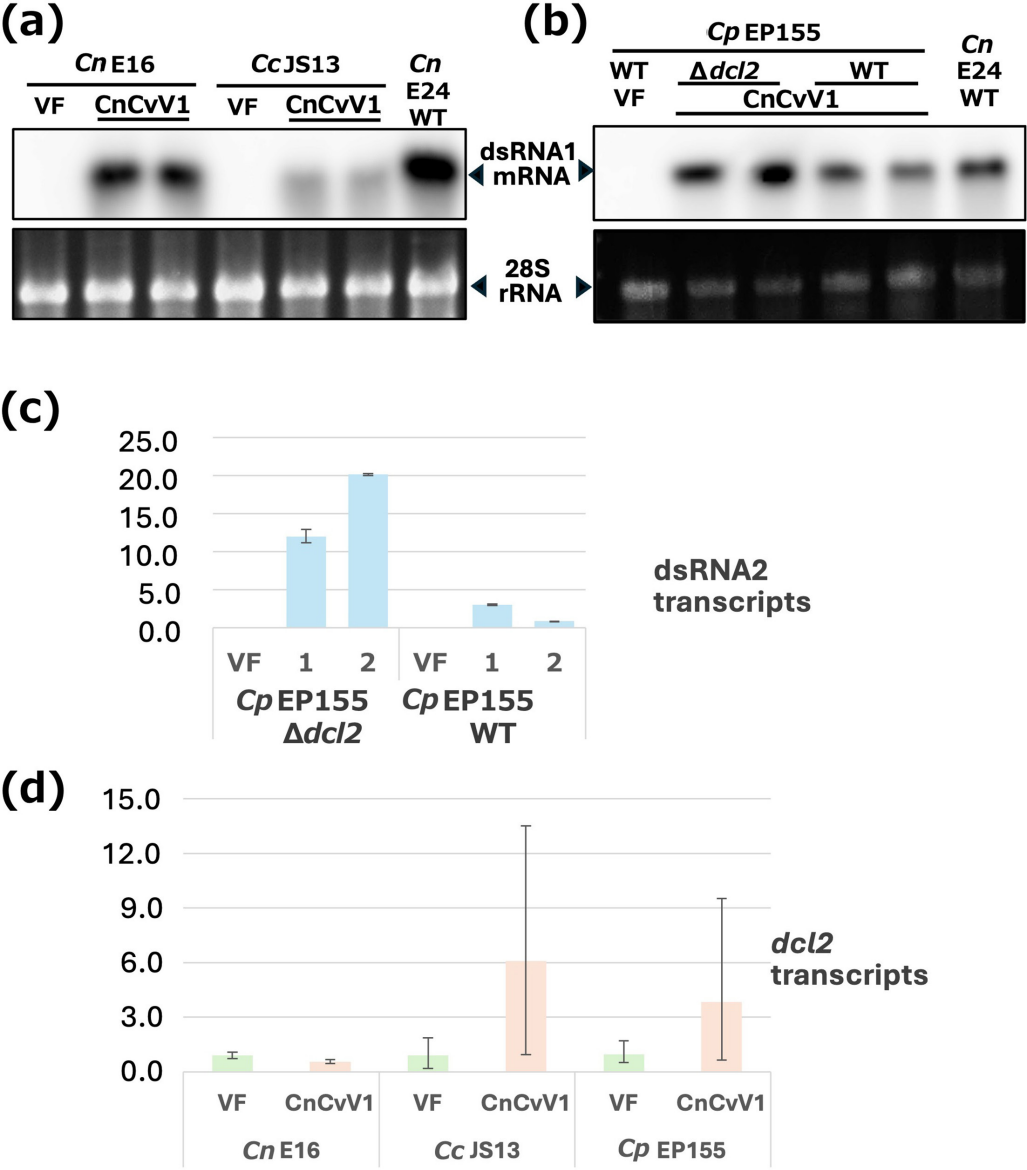
Comparison of CnCvV1 accumulation in different fungal strains. (**a**) CnCvV1 accumulation in different fungal host strains, *C. nitschkei* (*Cn*) E16 and *C. carpinicola* (*Cc*) JS13. The total RNA was prepared from CnCvV1-infected fungal strains obtained from protoplast fusion and subjected to northern blotting (**a, b**). Total ssRNA was probed with a digoxigenin (DIG)-labelled dsDNA fragment that corresponds to the CnCvV1 dsRNA1 mRNA. Ethidium bromide (EtBr)-stained 28S rRNA served as a loading control. The original CnCvV1-infected *C. nitschkei* E24 was used as a reference. (**b**) CnCvV1 accumulation in *C. parasitica* (*Cp*) EP155 (wt) and Δ*dcl2* (an RNA silencing-deficient mutant derived from EP155) as in (**a**). (**c**) CnCvV1 RNA was quantitatively compared by reverse transcription qPCR using two biological replicates. The sequences of the primer pairs used for actin mRNA (EP-g-actin-966F and EP-g-actin-1108R) and viral dsRNA2 transcripts (CnCvV1_RNA2-424F and CnCvV1_RNA2-546R) are shown in Table S1. Relative CnCvV1 transcript accumulation in *C. parasitica* wt strain replicate 2 is expressed as 1.0. (**d**) Transcriptional induction of *dcl2* upon CnCvV1 infection. *dcl2* expression was quantified by reverse transcription qPCR using the *actin* gene as an internal control using three biological replicates, as described by Sato *et al*. [44]. Relative *dcl2* mRNA accumulation in the virus-free (VF) is expressed as 1.0 in each fungal species. Student’s t-test revealed a statistically significant difference in transcript levels between the CnCvV1-free and CnCvV1-infected isogenic strains in *C. carpinicola* strain JS13 (*P*<0.01), but not in *C. parasitica* EP155.

We tested four fungal species (Table 1) for their ability to support CnCvV1 replication by using a protoplast fusion technique, in which we used the original CnCvV1-infected *C. nitschkei* E24 as the donor. The tested fungal species included *C. parasitica*, *C. nitschkei*, *C. carpinicola* and *V. ceratosperma*. Based on northern blotting, the three *Cryphonectria* species supported CnCvV1 replication (Table 2 and Fig. 5). However, we failed to infect *V. ceratosperma* with CnCvV1 despite repeated attempts to transfer the virus (Table 2). We confirmed the recipients’ genotypes with the PCR-based method described by Shahi *et al*. [26]. CnCvV1 was readily transferred between subcultures of the newly established host species via co-culturing. This observation further supports the horizontal transfer of CnCvV1 to the recipient strains. Interestingly, CnCvV1 accumulated much less (~10-fold) in the JS13 strain in *C. carpinicola* JS13 than in the original CnCvV1-infected *C. nitschkei* E24 or the experimentally infected *C. nitschkei* E16 that tended to allow a slightly lower CnCvV1 accumulation than strain E24 (Fig. 6a). Note that CnCvV1 accumulation in strain JS13 fluctuated considerably but did reach the level above the one exhibited by strains E16 and E24. This fluctuation might be associated with the observation that CnCvV1 was maintained unstably in *C. carpinicola* JS13 and eliminated during subculture and storage. There was a similar unstable maintenance in *C. parasitica* EP155. These phenomena contrast with the stable maintenance of CnCvV1 in the two *C. nitschkei* strains. We also applied another inoculation method, transfection, with crude virus fractions and sucrose gradient dsRNA-enriched fractions copurifying P2. A total of 96 *C*. *nitschkei* E16 subcultures regenerated after the transfection procedure tested negative. We obtained similar negative results with *C. carpinicola* JS13 and *C. parasitica* EP155 and Δ*dcl2* (data not shown).

**Table 2.** Horizontal transfer of CnCvV1 via protoplast fusion

Recipient		Donor	
		*C. nitschkei* LEP*-* E24/CnCvV1	
Species	Strain	Experiment I	Experiment II
*C. nitschkei*	E16	2/20 (10%)	1/20 (5%)
*C. parasitica*	EP155	16/20 (80%)	13/20 (65%)
*C. carpinicola*	JS13	8/20 (40%)	12/20 (60%)
*V. ceratosperma*	ASV53	0/20 (0%)	0/20 (0%)

Collectively, these results suggest that CnCvV1 can replicate in *C. parasitica*, *C. nitschkei*, and *C. carpinicola*, but not in *V. ceratosperma*, which belongs to the same order as *Cryphonectria* spp. (Diaporthales), but to a different family (Valsaceae).

### Asymptomatic infection of field isolates of host fungal species by CnCvV1

We investigated the possible effects of CnCvV1 infection on its host in different fungal strains that we newly established as virus hosts. We compared the phenotypes of a few pairs of isogenic virus-free and CnCvV1-infected *C. nitschkei* strains (the latter obtained via protoplast fusions). There were no discernible phenotypic differences between the CnCvV1-free and CnCvV1-infected *C. nitschkei* isolates (Fig. 5). We also observed symptomless infection in other fungal strains, namely *C. parasitica* EP155 and *C. carpinicola* JS13. Among the tested fungal strains, only *C. parasitica* Δ*dcl2* showed phenotypic alterations characterized by less aerial hyphal growth compared with the isogenic wt *C. parasitica* EP155. Therefore, we concluded that CnCvV1 does not induce symptomatic infection in any field-collected fungal strains that host the virus.

### CnCvV1 tolerates host antiviral RNA silencing

We horizontally transferred CnCvV1 to RNA silencing-deficient *C. parasitica* Δ*dcl2*, which has the standard EP155 background for which many mutants and molecular tools are available [19]. In an effort to examine the effects of antiviral RNA silencing on CnCvV1, we compared CnCvV1 accumulation between EP155 and *C. parasitica* Δ*dcl2*. We observed a 2–10-fold decrease in the CnCvV1 content in *C. parasitica* EP155 or *C. nitschkei* relative to *C. parasitica* Δ*dcl2*, based on the intensity of the CnCvV1 RNA1 band on a northern blot (Fig. 6b). We confirmed this result by performing reverse transcription qPCR with independent subcultures (Fig. 6c).

In fungi, RNA silencing key genes are often upregulated transcriptionally [22,52, 53]. We compared the *dcl2* transcript level between CnCvV1-free and CnCvV1-infected isogenic strains of different *Cryphonectria* spp. by using reverse transcription qPCR. We found that *dcl2* was upregulated 3–6-fold in *C. carpinicola* JS13 or *C. parasitica* EP155 upon infection by CnCvV1. In contrast, *dcl2* expression did not change in *C. nitschkei* E16 following infection by CnCvV1 (Fig. 6d). A statistically significant difference in transcript levels between the CnCvV1-free and CnCvV1-infected isogenic strains was observed in *C. carpinicola* strain JS13 (*P*<0.01), but not in *C. parasitica* EP155.

## Discussion

This study represents a thorough characterization of a novel curvulavirid (order *Durnavirales*), CnCvV1, detected from a Japanese strain (E24) of *C. nitschkei* (Figs1  2). There is limited information available for curvulavirids in general, despite the growing genomic sequence database. We also sequenced another isolate of CnCvV1 from a different strain (E49) of *C. nitschkei*. CnCvV1 is closely related to other curvulavirids, particularly to Botryosphaeria dothidea bipartite mycovirus 1 and CpBMV1. Note that CnCvV1 shares over 90% nucleotide sequence identity with CpBMV1 (92.8% for dsRNA1 and 93.0% for dsRNA2), whose genome sequence has been deposited in the GenBank/EMBL/DDBJ databases (accession numbers KC549809 and KC549810). No other properties have been reported for CpBMV1, which may be the same virus described previously as a partiti-like RNA from *C. parasitica* [54]. Phylogenetic and sequence analyses (Fig. 3) indicated the placement of CnCvV1 and CpBMV1 into the same species within the family *Curvulaviridae*, which currently has only one genus (*Orthocurvulavirus*) [2].

Heterogeneity in genome organization has been noted for curvulavirids. CThTV dsRNA1 has two ORFs [8], while many other curvulavirids possess only a single continuous ORF that encodes RdRP. It should also be noted that the deletion of a nucleotide at map position 1,227 or 1,278 of the positive-strand of CThTV dsRNA1 would lead to the creation of one single ORF and translation of the conserved amino acid sequence stretch (--APICRDDGIRL--). In addition, dsRNA2 encodes either one or two ORFs. The ORF configuration is not related to phylogenetic placements of curvulavirids. CnCvV1 dsRNA1 has a single large ORF corresponding to 80% of the entire segment size, while CnCvV1 dsRNA2 has two ORFs that encode a protein tightly associated with its genomic dsRNA (Fig. 2a) and a small protein of unknown function. The dsRNA2 ORF2-encoded proteins, conserved among curvulavirids, show only moderate levels of amino acid sequence identity. Although 3′-proximal small ORFs of dsRNA2 have not always been documented in the databases or publications, they are well conserved in curvulavirids. The small ORFs are situated in the same frame as or in frames different from the preceding large capsid protein-encoding ORFs. This fact strongly suggests the expression of the small ORFs, although how they are expressed remains unknown.

There is scarce biological information about curvulavirids because neither infectious entity nor experimental virus introduction protocols have been established for most of these viruses. Thus, a cause-and-effect relationship has rarely been established between curvulavirids and their hosts. There are only two exceptions: CThTV and HetRV6. CThTV is the best characterized curvulavirid: it forms isometric particles that are ~27 nm in diameter and can be laterally transferred to virus-free host fungi via coculturing [8]. CThTV can confer heat tolerance to its fungal host and the plant host of the host fungus. The within-species diversity and phenotypic effects on host fungi have been well investigated for another curvulavirid, HetRV6 [10,12]. This virus has both positive and negative impacts on its host fungi, depending on the species and the environmental conditions. In the present study, we adopted a protoplast fusion protocol [26] to introduce CnCvV1 into host candidate fungi. CnCvV1 is the third example of curvulavirids in which biological properties are available, and its current characterization is significant for several reasons. By comparing a few sets of virus-free and virus-infected isogenic fungal strains, we could conclude that CnCvV1 results in asymptomatic infections in the tested species (i.e. *C. parasitica*, *C. nitschkei* and *C. carpinicola*) (Fig. 5), unlike CThTV and HetRV6. Of note, *C. parasitica* Δ*dcl2*, an RNA silencing-deficient mutant, showed symptomatic infection (reduced growth rate), as in many cases, where this fungal strain allows for higher levels of virus accumulation and shows symptomatic infection. The currently established protofusion method for the experimental inoculation of CnCvV1 will be useful for the biological characterization of other curvulavirids.

A few laboratories have conducted similar experiments to determine the experimental host range of different viruses, such as a mitochondrially replicating capsidless (+)RNA mitovirus (order *Cryppavirales*) [26], capsidless (+)RNA hypovirus [32,55], encapsidated dsRNA partitivirus (order *Durnavirales*) [56], chrysovirus [32] and pseudototivirus (order *Ghabrivirales*) [57]. Similar to CnCV1 (a chrysovirus) [32], CnCvV1 appears to have a relatively narrow host range – that is, only members of the genus *Cryphonectria*. Note that Cryphonectria hypovirus 1 (CHV1, a hypovirus), Rosellinia necatrix victorivirus 1 (RnVV1)/Helminthosporium victoriae virus 190S (victoriviruses) and Rosellinia necatrix partitivirus 6 (partitivirus) can experimentally cross and replicate in different subclasses [26,32, 47, 57,59]. Cross-kingdom replication of CHV1 and fungal partitiviruses has recently been reported [60]. However, no such information is available for other curvulavirids. HetRV6 is the only exception: it has been shown to undergo interspecies horizontal transmission [12], as for other mycoviruses [27,61,65]. We found that several *Cryphonectria* species can host CnCvV1 (Table 2, Figs5  6). CnCvV1 was experimentally shown to replicate in *C. parasitica*, *C. carpinicola* and *C. nitschkei. C. nitschkei* is sympatric to *C. parasitica* on chestnut trees or closely related species in the family Fagaceae. Thus, it is not surprising that different strains of a single virus species are naturally isolated from the two *Cryphonectria* species, possibly due to natural horizontal CnCvV1 transfer in the same niche. Milgroom *et al*. [61] reported the lateral interspecies transfer of the prototype hypovirus CHV1 under laboratory and natural conditions between different *Cryphonectria* species. We have also clearly indicated experimental horizontal transmission of a chrysovirus, a mitovirus and a fusagravirus (order *Ghabrivirales*) between different *Cryphonectria* species [26,27, 32]. Collectively, these observations support naturally occurring horizontal interspecies transfer of fungal viruses.

The chestnut blight fungus, *C. parasitica*, is a model filamentous host for studying virus–host interactions [19], particularly for exploring host antiviral RNA silencing and viral counter-defence [66]. Many fungal viruses, whether homologous or heterologous, that can replicate in *C. parasitica* are targeted by RNA silencing (or RNAi) in this fungus. In general, RNA silencing key genes are induced transcriptionally upon virus infection and function against viruses in fungi [21,22, 24, 25, 52]. Virally encoded RNA silencing suppressors (RSSs) often impair the transcriptional upregulation as a counter-defence [22,23, 67, 68]. However, how a virus is susceptible or tolerable to antiviral RNA silencing varies depending on the virus and may be related to viral counter-defence. For example, the accumulation levels of some viruses such as RnVV1 (a victorivirus), Rosellinia necatrix mycoreovirus 2 (MyRV2) (a mycoreovirus), Rosellinia necatrix megabirnavirus 1 (a megabirnavirus) and an RSS-lacking mutant of CHV1 (CHV1-Δp69) are greatly augmented, ~20-fold or above, in RNA silencing-deficient mutants [44,58, 69]. Interestingly, RnVV1 and MyRV2 are susceptible to Dicer-dependent, Ago-independent antiviral defence [44]. Other viruses, such as the prototype hypovirus CHV1, a betapartitivirus RnPV6 (Rosellinia necatrix partitivirus 6) and a mycoreovirus MyRV1 (mycoreovirus 1), show slightly increased accumulation in *C. parasitica* Δ*dcl2* [47,70]. Therefore, the observation that CnCvV1 accumulated more in Δ*dcl2* than in *C. parasitica* EP155 was not that surprising. Whether RNA silencing interferes with the replication of a fungal virus may be related to how it is perceived by its host and induction of RNA silencing, as well as the strength of RNA silencing suppression by the virus. Little is known about the RSSs of curvulavirids. Viruses with strong RSS often induce severe symptoms, and transgenic expression of them causes phenotypic alterations, as exemplified by CHV1 [70,71]. However, CnCvV1 showed asymptomatic infection in *C. nitschkei*, given that it failed to induce transcription of *dcl2*. These findings suggest that CnCvV1 inhibits the upregulation of RNA silencing key genes or can evade perception by host defence mechanisms.

 Virion transfection is a convenient method to experimentally introduce encapsidated fungal viruses, particularly dsRNA viruses, as exemplified by partitiviruses, which are phylogenetically related to curvulavirids [58,72]. An unexpected result in this study was our failure to purify rigid virus particles that we could use for transfection. Although the genomic dsRNA and P2 (p36) of CnCvV1 cofractionated in a sucrose gradient, we did not observe rigid particles. One explanation for this outcome is that CnCvV1 is unable to form rigid particles, similarly to a dsRNA polymycovirus, Aspergillus fumigatus tetramycovirus 1 (AfuTmV1, a polymycovirid), which makes a genomic dsRNA–protein complex without forming rigid particles [8]. Consistently, the RNase assay showed an indistinguishable profile between CnCvV1 (a curvulavirid) and PjPmV1 (a polymycovirid) (Fig. 4). It is possible that CnCvV1 particles are unstable during purification and subsequent electron microscopy (EM). Further experimentation, such as EM of ultrathin sections of freeze-fried mycelia or direct EM of mycelial homogenates, is required to determine the morphology and infectious entity of CnCvV1. Virion transfection of another curvulavirid, CThTV, which was confirmed to form isometric particles, has not been reported.

## Supplementary material

10.1099/jgv.0.002177 Uncited Supplementary Material 1.

## Abbreviations

BdBV1: Botryosphaeria dothidea bipartite mycovirus 1
CHV1: Cryphonectria hypovirus 1
CnCvV1: Cryphonectria nitschkei curvulavirus 1
CpBMV1: Cryphonectria parasitica bipartite mycovirus 1
CThTV: Curvularia thermal tolerance virus
DeBV1: Diaporthe eres bipartite mycovirus 1
DIG: digoxigenin
EM: electron microscopy
HetRV6: Heterobasidion RNA virus 6
MAFF: Ministry of Agriculture, Forestry and Fisheries
MyRV2: Rosellinia necatrix mycoreovirus 2
NCBI: National Center for Biotechnology Information
NGS: next-generation sequencing
PDA: potato dextrose agar
PDB: potato dextrose broth
qPCR: quantitative PCR
RdRP: RNA-dependent RNA polymerase
RLM-RACE: RNA ligase-mediated rapid amplification of cDNA ends
RnVV1: Rosellinia necatrix victorivirus 1
RSSs: RNA silencing suppressors
