# Supplementary material for: Thorough characterization of a new curvulavirid from a Japanese strain of Cryphonectria nitschkei
Source: J Gen Virol. 2025 Dec 17;106(12):002177. doi: 10.1099/jgv.0.002177 (PMC12711003; doi:10.1099/jgv.0.002177)
Supplement: Uncited Supplementary Material 1. [file jgv-106-02177-s001.pdf]

Thorough characterization of a new curvulavirid from a Japanese strain of *Cryphonectria nitschkei*

Sabitree Shahi[1+], Sakae Hisano[1], Wasiatus Sa'diyah[1++], Yoshihiro Takaki[2], Hideki Kondo[1], and Nobuhiro Suzuki[1,3*]

[1]Institute of Plant Science and Resources, Okayama University, Kurashiki, Okayama 710-0046[1)], Japan
[2]Institute for Extra-cutting-edge Science and Technology Avant-garde Research (X-star), Japan Agency for Marine-Earth Science and Technology (JAMSTEC), Yokosuka, Japan.
[3]Neo-Virology Laboratory, Graduate School of Agricultural Science, Tohoku University, Sendai, Miyagi 980-8572, Japan

Short title: A curvulavirid with a narrow host range

*Correspondence may be sent to N. Suzuki
IPSR, Okayama University, Chuou 2-20-1, Kurashiki, JAPAN
Telephone: 81-86-434-1230
FAX: 81-86-434-1232
E-mail: nsuzuki@okayama-u.ac.jp

Present address: +Liver Diseases Branch, NIDDK, National Institutes of Health, Bethesda, MD 20892-1800; ++Laboratory of Ecosystem and Coevolution, Graduate School of Biostudies, Kyoto University, Kyoto, Japan

Manuscript information:
Abstract, 175 words; Text, 4,941 words excluding figures legends and references; Figures, 6; Tables, 2; Supplementary Figure, 1: Supplementary Table, 1
Keywords: *Cryphonectria nitschkei*; *Cryphonectria parasitica*; *Cryphonectria carpinicola*; curvulavirus; fungal virus; dsRNA; host range, RNA silencing

DATA AVAILABILITY

The complete nucleotide sequences of two CnCvV1 strains in this article have been deposited with the EMBL/GenBank/DDBJ Data Library under Accession Nos. LC781670 and LC781671, and LC781672 and LC781673.

Supplementary figure legends

Fig. S1. Separation of the CnCvV1 virus fractions by centrifugation. (**a**, **b**) Crude mycelial extracts from CnCvV1-infected *Cryphonectria nitschkei* E24 were fractionated via conventional sucrose gradient centrifugation (**a**) or CsCl equilibrium gradient centrifugation (**b**). Each fraction was tested for its dsRNA and protein component by 1% agarose gel electrophoresis using 1× TAE (**top panels** in **a** and **b**) or 10% sodium dodecyl sulfate–polyacrylamide gel electrophoresis (SDS-PAGE; **bottom panels** in **a** and **b**). Viral dsRNA and protein p36-containing fractions are denoted by red lane numbers, with peak fractions in bold. Fractionation positions of a partitivirus (Rosellinia necatrix partitivirus 11, RnPV11) and a victorivirus (Helminthosporium victoriae virus 190S, HvV190S) in the respective gradient centrifugation are indicated by arrows at the bottom of the SDS-PAGE gels. Lane M is the size standard electrophoresed in parallel: 1-kb DNA ladder (Thermo Fisher) (**top panels** in **a** and **b**) or the Precision Plus protein Dual Color standards (Bio-Rad).

Fig. S1

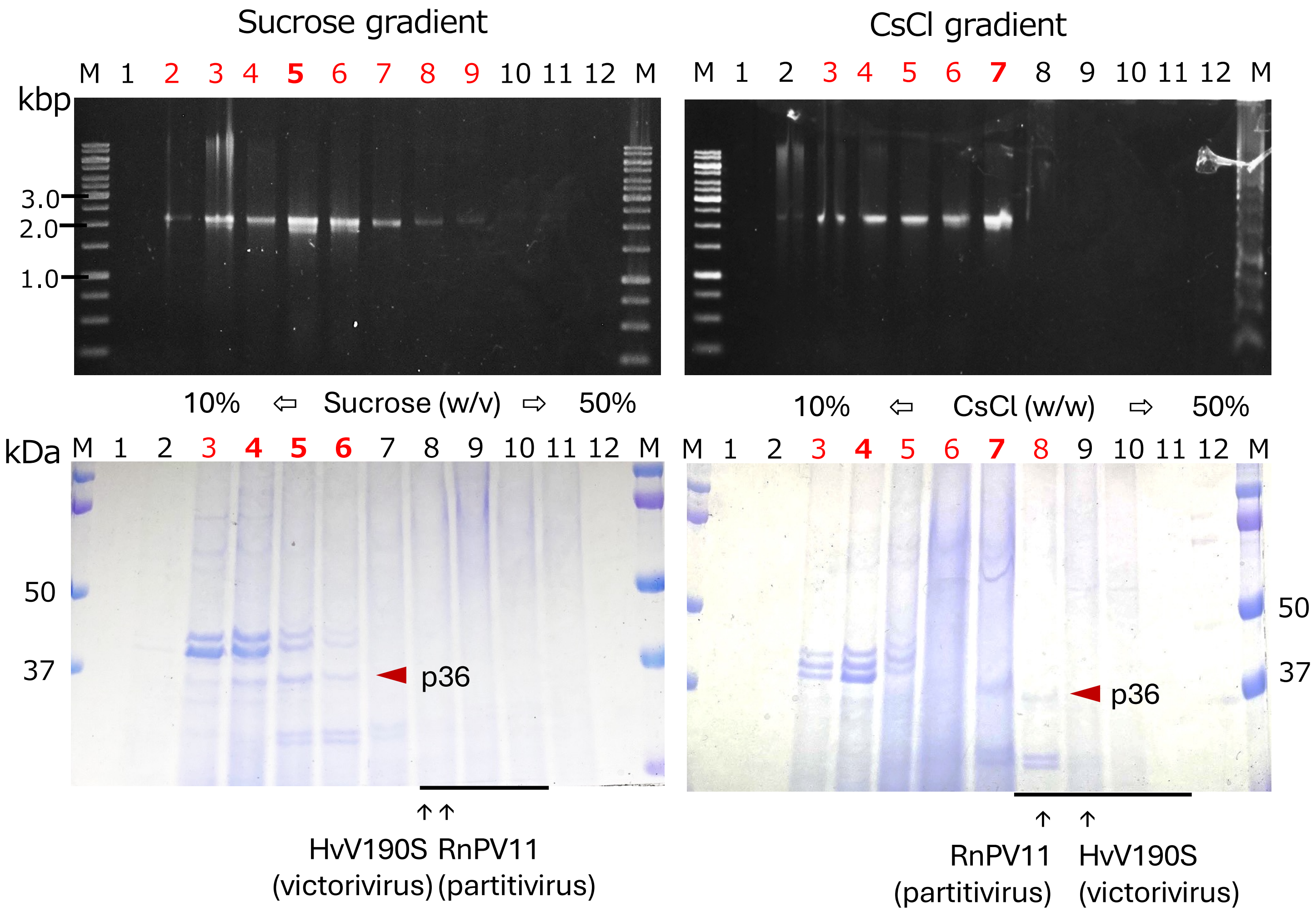

Sucrose gradient
CsCl gradient
M 1 2 3 4 5 6 7 8 9 10 11 12 M
kbp
3.0
2.0
1.0
10% ⇦ Sucrose (w/v) ⇨ 50%
10% ⇦ CsCl (w/w) ⇨ 50%
kDa
50
37
p36
HvV190S RnPV11
(victorivirus) (partitivirus)
RnPV11 HvV190S
(partitivirus) (victorivirus)

Table S1. Primers List

Primer name	Sequence	Purpose	Target	Position / Note		
CnCvV1_RNA1_RACE-R	AAATTTGCCAGGGTAGTAACGC	RACE	CnCvV1-RNA1	604	<	625
CnCvV1_RNA1_RACE-F	CACTGGTCTGGCAAGAAGTCCTT	RACE	CnCvV1-RNA1	1412	>	1434
CnCvV1_RNA2_RACE-R	GAGTTTACGCGCTGTCGTCTG	RACE	CnCvV1-RNA2	231	<	251
CnCvV1_RNA2_RACE-F	ATACGAGCATGGAGGAAATGGAC	RACE	CnCvV1-RNA2	1180	>	1202
CnCvV1_RNA1_Probe-F	TACTACCCTGGCAAATTTGCTGGCA	Northern	CnCvV1-RNA1	608	>	632
CnCvV1_RNA1_Probe-R	GATCTGGAAGGCATCCCACATTACG	Northern	CnCvV1-RNA1	1387	<	1411
CnCvV1_RNA1-854F	CTTAAGTTATGTGGAGCGCAC	RT-PCR	CnCvV1-RNA1	854	>	873
CnCvV1_RNA1-1240R	GATGAGGGTTATGATCGACTGTA	RT-PCR	CnCvV1-RNA1	1218	<	1240
CnCvV1_RNA2-231F	CAGACGACAGCGCGTAAACTC	RT-PCR	CnCvV1-RNA2	231	>	251
CnCvV1_RNA2-1202R	GTCCATTTCCTCCATGCTCGTAT	RT-PCR	CnCvV1-RNA2	1180	<	1202
CnCvV1_RNA2-527F	GGTGGCAGAGAACGCTATGTTC	RT-PCR	CnCvV1-RNA2	527	>	548
CnCvV1_RNA2-840R	CATTAATGGGATCTTTCTTCGGC	RT-PCR	CnCvV1-RNA2	818	<	840
CnCvV1_RNA2-424F	ACTTCGTCACCAACCGAGAG	RT-qPCR	CnCvV1-RNA2	424	>	443
CnCvV1_RNA2-546R	ACATAGCGTTCTCTGCCACC	RT-qPCR	CnCvV1-RNA2	527	<	546
EP-g-actin-966F	GCCATTACGAACAGTATCCAGCGG	RT-qPCR	Host mRNA	Common*: Cn, Cc, Cp		
EP-g-actin-1108R	GTGACAGCCTCCTTGATGTGCCG	RT-qPCR	Host mRNA	Common*: Cn, Cc, Cp		
EP-g-dcl2-2119F	GTTCGCCCTGGAATGAGGGAAGT	RT-qPCR	Host mRNA	Common*: Cn, Cc, Cp		
EP-g-dcl2-2297R	GGCTTCATCTCAGGCAGAGCA	RT-qPCR	Host mRNA	Common*: Cn, Cc, Cp		
EP-m-dcl2-1996F	TGGGTGTCTGAAAAGAATGCGTGC	sequencing	Host mRNA	Common*: Cn, Cc, Cp		
EP-m-dcl2-2754R	TGGTCGGTGGAAGAAGCCTGT	sequencing	Host mRNA	Common*: Cn, Cc, Cp		

*These primers work well commonly for three fungal species: *Cryphonectria nitschkei* (Cn), *C. carpinicola* (Cc), and *C. parasitica* (Cp).
